# Spatial congruence between multiple stressors in the Mediterranean Sea may reduce its resilience to climate impacts

**DOI:** 10.1038/s41598-018-33237-w

**Published:** 2018-10-05

**Authors:** Francisco Ramírez, Marta Coll, Joan Navarro, Javier Bustamante, Andy J. Green

**Affiliations:** 1 Departament de Biologia Evolutiva, Ecologia i Ciències Ambientals, Universitat de Barcelona, Av. Diagonal 643, 08028 Barcelona, Spain; 2 Department of Wetland Ecology, Estación Biológica de Doñana (CSIC), C/Américo Vespucio 26, 41092 Sevilla, Spain; 3 Institut de Ciències del Mar (ICM-CSIC), Passeig Maritim de la Barceloneta, 37-49, 08003 Barcelona, Spain; 4 Remote Sensing and GIS Lab (LAST-EBD). Estación Biológica de Doñana (CSIC), C/Américo Vespucio 26, 41092 Sevilla, Spain

## Abstract

Climate impacts on marine ecosystems may be exacerbated by other, more local stressors interacting synergistically, such as pollution and overexploitation of marine resources. The reduction of these human stressors has been proposed as an achievable way of retaining ecosystems within a “safe operating space” (SOS), where they remain resilient to ongoing climate change. However, the operability of an SOS requires a thorough understanding of the spatial distribution of these climate and human impacts. Using the Mediterranean Sea as a case study, we illustrate the spatial congruence between climate and human stressors impacting this iconic “miniature ocean” synergistically. We use long-term, spatially-explicit information on the distribution of multiple stressors to identify those highly impacted marine areas where human stressors should be prioritized for management if the resilience to climate impacts is to be maintained. Based on our spatial analysis, we exemplify how the management of an essential supporting service (seafood provision) and the conservation of a highly impacted Mediterranean sub-region (the Adriatic Sea) may benefit from the SOS framework.

## Introduction

There is now overwhelming evidence for human-induced climate change on a scale that may result in increasing extinction rates or ecosystem collapses^1,2^. Given this scenario, it has been proposed that ecosystem resilience to climate change should be improved through local pro-active management^3–6^. Indeed, critical climate levels for ecosystem collapse may be influenced by conditions that can be managed locally^4,7,8^. Thus, reducing local stressors may keep ecosystems within a “safe operating space” (SOS, sensu^9^), where they remain resilient to climate change^10^. The practicality and scale of such local measures makes them more feasible than global greenhouse gas management^6,10^.

There is virtually no part of the global environment that we have left “unchanged”. However, the oceans are of particular concern since they are among the most complex, difficult to study, poorly understood and most impacted of Earth’s biomes^1,11^. Among the suite of human stressors impacting marine ecosystems, the most alarming threats are ocean pollution and overfishing^11–14^, and their effects appear to increase the sensitivity of ecosystems to climate impacts such as ocean warming and acidification^15,16^. Accordingly, climate and human stressors should not be treated in isolation from each other when developing conservation and management strategies for marine ecosystems^4,17^.

Spatially-explicit assessments on the distribution of climate and human impacts are vital for identifying marine areas of particular concern that are simultaneously impacted by different stressors^5,15,17,18^. This may represent a major challenge for marine conservation today, given the scarcity of data with the necessary spatiotemporal resolution. Fortunately, satellite remote-sensing data now provide a means for studying the most recent and striking trends and patterns in physical (e.g., sea surface temperature, SST), chemical (e.g., N, P and O_2_ concentrations) and biological (e.g., marine productivity) variables in the world’s oceans at unprecedented spatio-temporal resolutions^3,5,11^. Recent advances in modelling techniques based on Automatic Identification Systems (AIS) track data for fishing vessels are also providing more reliable information on the spatial distribution of fishing effort^19^. Together, these data provide a uniquely detailed insight into the spatio-temporal distribution of climate and additional human stressors threatening marine communities.

**Figure 1:**
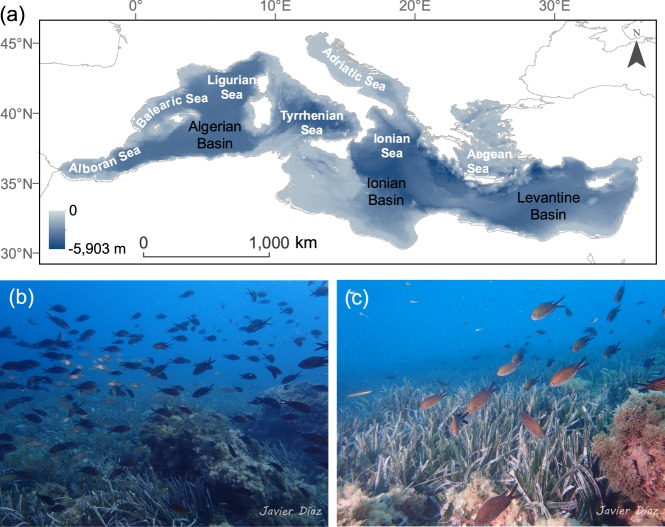
The Mediterranean Sea: a “miniature ocean” and a “giant mesocosm”. The Mediterranean Sea (**a**) is the deepest enclosed sea on Earth. Located between Africa, Europe and Asia, it connects with the Atlantic and the Indian Ocean through the Strait of Gibraltar and the Suez Canal. The Strait of Sicily divides the sea into two sub-basins with contrasting oceanographic conditions, the western (0.85 million km^2^) and the eastern (1.65 million km^2^). The Mediterranean Sea hosts a number of emblematic species and habitats, such as the seagrass meadows of *Posidonia oceanica* (**b**, **c**) (images courtesy of Javier Díaz). Because of its particular oceanographic, geological and biological conditions, this “miniature ocean” is a giant mesocosm that can be used for horizon-scanning assessments on how the Earth’s oceans are responding to multiple stressors^7^.

Here, by combining information on the spatial distribution of these multiple stressors, we aimed to identify highly impacted marine areas for the Mediterranean Sea (Fig. 1), where the relatively “easy-to-handle” local and medium-scale stressors require management within a SOS framework to enhance resilience to climate change. In particular, we combined long-term remote-sensing data with data on the spatial distribution of fishing pressure to identify areas simultaneously impacted by climate impacts, diffuse sources of pollution and industrial fisheries. Spatially explicit information on the impact of climate change was derived by combining long-term data of SST (1982–2016) with ocean pH and CO_2_ partial pressure (*p*CO_2_, 1999–2016). Changes in ocean biochemistry, likely driven by land-based pollution sources, were mapped by developing a spatially-explicit index that combined long-term (1999–2016) information on nutrient (phosphate and nitrate) concentrations, net primary production (NPP) and dissolved oxygen concentrations (dO_2_). Finally, available time series (2012–2017) of data on the spatial distribution of fishing pressure^19^ were used as a proxy for local impacts of human fisheries on marine ecosystems. We follow up this spatial analysis by exemplifying the potential value of our spatially-explicit assessments in the management of a thermophobic (i.e. heat intolerant) fish with high commercial value that is showing signs of collapse in the Mediterranean (European sardine, *Sardina pilchardus*)^20^; and the conservation of the Adriatic Sea, a well-known highly impacted sub-region where pollution and overexploitation have been intense^21^.

## Results and Discussion

### Trends and patterns for interacting stressors

**Figure 2:**
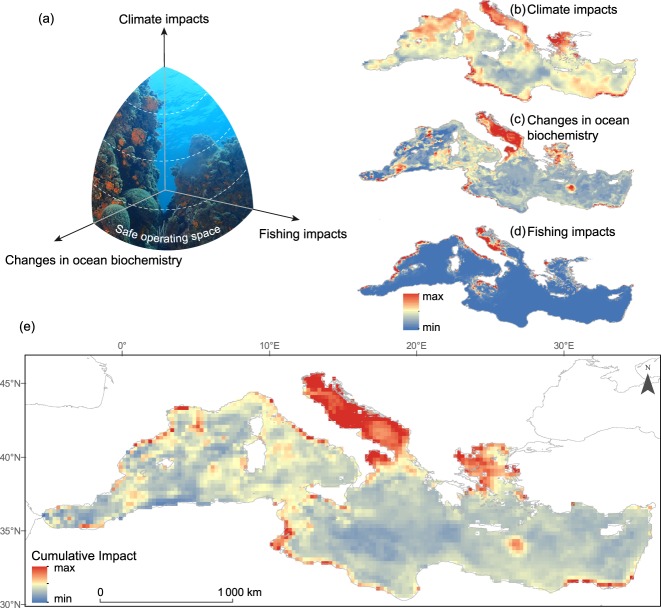
Spatial congruence of climate and human impacts: a SOS for the Mediterranean Sea. Three dimensions of the safe operating space (SOS) for the Mediterranean Sea (**a**) (image courtesy of Javier Díaz), reflecting the intensity of (**b**) climate change-induced ocean warming (sea surface temperature, CO_2_ partial pressure and pH), (**c**) changes in ocean biochemistry likely caused by changes in land-use (nutrient concentrations, net primary production and dissolved oxygen concentrations), and (**d**) fishing impacts. All these stressors are combined in (**e**) to provide an overview of the spatial heterogeneity in the magnitude of environmental changes and highlight those marine areas that have undergone the largest recent changes in environmental conditions. Maintaining these systems in a desirable state of conservation and sustainable use (by retaining them within the safe operating space) as climate change progresses requires management of interacting human stressors to bring them down from excessive levels (indicated by the lower arcs in dashed, blue lines in -a-) to acceptable ones (upper arcs). Adapted from Fig. 3 in^6^.

Our spatially-explicit assessments on the cumulative impact of climate change (SST, *p*CO_2_ and pH), land-based pollution (nitrate and phosphate concentration, NPP and dO_2_) and commercial fisheries, reveal spatial overlap of these multiple stressors in coastal and shelf areas across the whole Mediterranean Sea (Fig. 2). The most impacted marine zones occur through coastal and shelf areas from the Balearic, Tyrrhenian, Adriatic and Aegean Seas in the North; and the Central and Eastern African coast in the South. These general trends and patterns are consistent when considering climate and human impacts separately (Fig. 2).

Climate impacts, as indicated by the long-term trends in SST, *p*CO_2_ and pH, are unevenly distributed within the Mediterranean Sea (Fig. S1), and broadly concur with the more general and global trends towards increasing water temperatures, CO_2_ concentrations, and seawater acidification^1,3,11^. However, there is much spatial heterogeneity in the magnitude of these changes. Increasing trends in SST are particularly acute in the Eastern Mediterranean (Fig. S1a,b), whereas the sharpest changes in ocean pH and *p*CO2 have occurred in the Western and Central Mediterranean, particularly along the Central and Eastern African coasts and within the Adriatic Sea (Fig. S1d–i).

Our results indicate that coastal and shelf areas of the Mediterranean Sea are most impacted by pollution, eutrophication or anoxia events. These highly impacted areas mainly occur in the Adriatic and the Aegean Seas, but also along the Central and Eastern African coasts (Figs 2 and S2), where runaway population growth is creating unprecedented anthropic pressure on marine ecosystems^7^.

Finally, our fine-scale analysis of fishing pressure revealed that fishing effort is particularly acute over the continental shelfs and in the North-Western and Central Mediterranean, where most overexploited commercial species occur^22^. This includes regions within the Alboran and the Balearic Seas, but also within the Ligurian, Tyrrhenian and Adriatic Seas (Fig. 2).

### Climate and human impacts on sardine stocks

**Figure 3:**
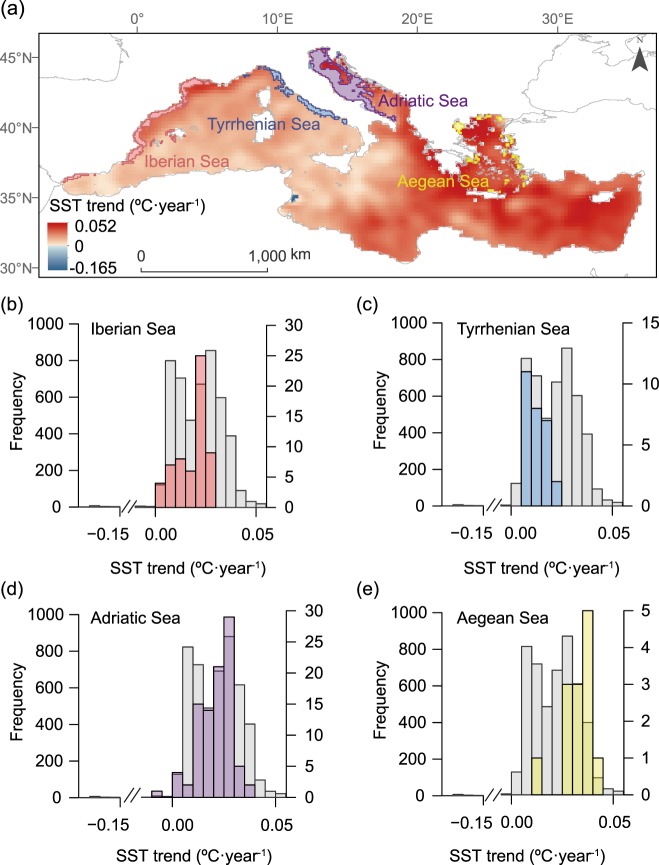
Ocean warming and industrial fisheries: two stressors impacting fish stocks synergistically. Spatial overlap between the distribution of fishing pressure and the long term (1982–2016) trend in mean winter values (from January 1^st^ to March 31^th^) of sea surface temperature -SST- (**a**). Fishing pressure is particularly high at four different coastal areas (each marked in a different color; fishing pressure >4^th^ quartile; from East to West): (**b**) the Iberian coast, (**c**) Tyrrhenian Sea, (**d**) Adriatic Sea and (**e**) Aegean Sea. The histograms represent the frequency distribution of estimated slopes for pixel-based long-term trends (linear regressions in °C·year^−1^) of winter SST for the whole Mediterranean Sea (in grey, left y-axis) and for different fishing hotspots (colored bars, right y-axis).

The combination of ocean warming and fishing pressure may represent an “allied attack” on heat -intolerant fish species with high commercial value^17,20^. This could be the case for the European sardine. Increasing water temperature, particularly during winter when this species reproduces, may decrease breeding performance and, potentially, cause population declines^20^. Climate-driven changes in the distribution of competitive or invasive fish species (e.g. round sardinella *Sardinella aurita*) may complicate this picture^23^. The European sardine is among the most important target species for commercial fisheries in the Mediterranean^24^ and historically has been one of the most abundant species in the ecosystem. Sardine is currently overexploited and shows very low population levels^25^. Our assessments suggest that those areas with the highest fishing pressure have also undergone increasing water temperatures during the winter period (from January to March, Fig. 3). Fishing pressure may have therefore exacerbated climate impacts on this small pelagic fish. The European sardine is, in turn, a key species linking bottom-up and top-down processes in the Mediterranean^26^. Changes in its abundance can affect the structure and functioning of the whole ecosystem and, ultimately, essential supporting services (seafood provision). Based on the SOS framework, fishing policies that promote sustainable fishing practices or the spatial redistribution of fisheries are required to allow sardine populations to withstand climate change impacts. This is urgent in areas where temperature has increased the most. Given that different fishing gear target different commercial species^27^, additional gear-specific, spatially-explicit information on fishing effort would also be necessary in order to draw firm conclusions on the cumulative impact of climate change and commercial fisheries on this, or any other particular fish species.

### Interacting stressors in the Adriatic Sea

**Figure 4:**
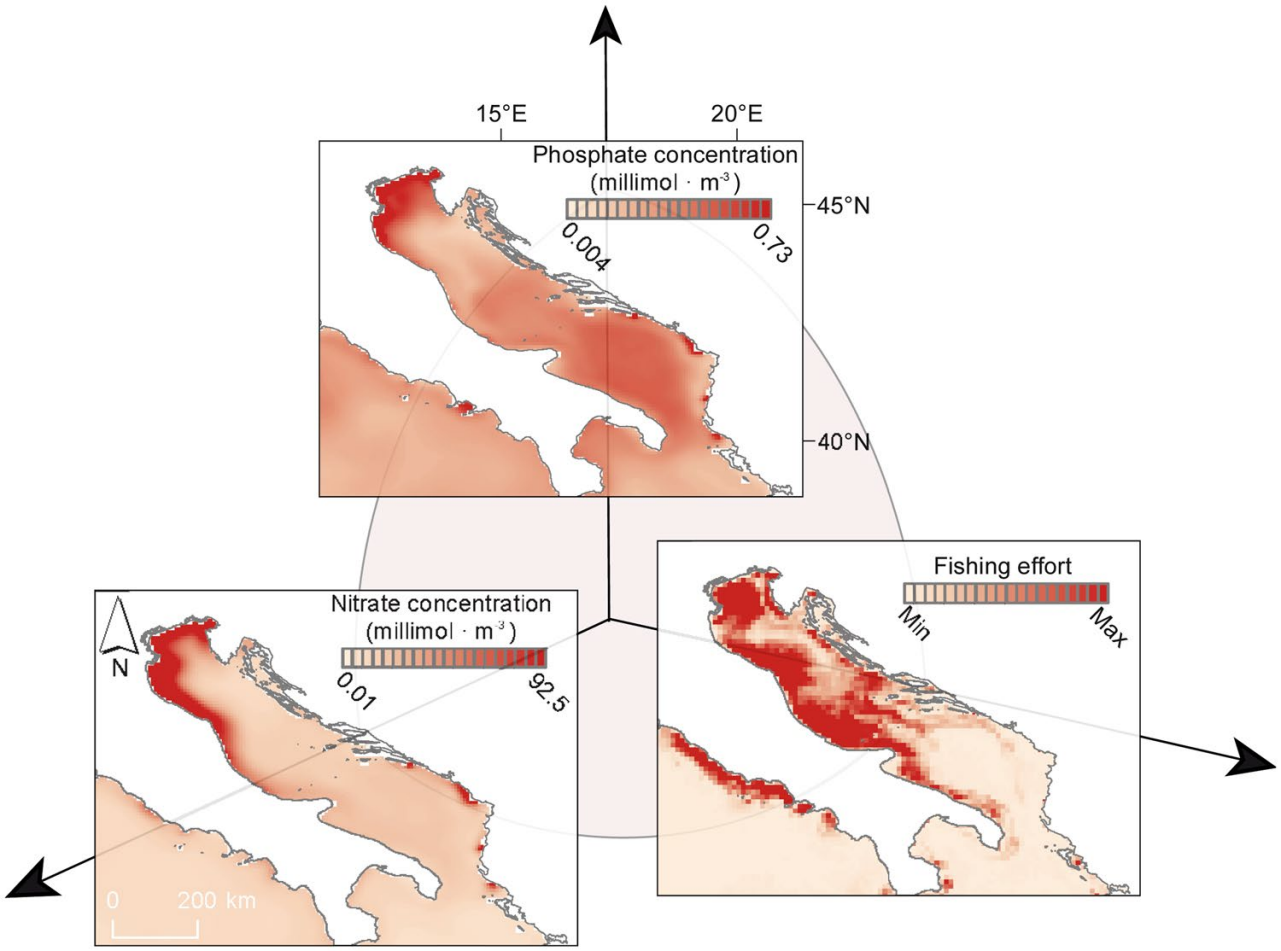
Main stressors interacting synergistically within the Adriatic Sea, which exemplify the axes in an SOS. Fine-scale spatial distribution for three major environmental stressors impacting the Adriatic Sea: industrial fishing pressure and nitrate and phosphate concentration (mean value for the 1999–2016 period). Despite the decreasing trend in nitrate concentrations observed for this area (see Fig. S2), the Northern Italian coast still shows relatively high nitrate and phosphate concentrations. This area is also heavily impacted by commercial fisheries. Together, these stressors are likely to synergistically reduce ecosystem resilience to climate change.

With a long history of human-induced changes in habitats, water quality and marine biodiversity, the Adriatic Sea represents a classic example of synergistic impacts affecting Mediterranean ecosystems^21,28^. Today, 98% of traditional marine resources are depleted to less than 50% of former abundance, with a large number of species that have become functionally extinct^21,29^. Species providing habitat and filtering functions have been reduced by 75%, exacerbating the degradation of water quality and increased eutrophication^21^. The increasing human population in the surrounding areas, along with land reclamation, deforestation and agriculture, resulted in increasing nutrient loads and eutrophication during the 80 s^30,31^. Although some ecosystem processes (e.g. productivity or total fish biomass) may benefit from moderate nutrient inputs, human-driven eutrophication is a pervasive and widespread process negatively impacting marine species inhabiting, and hence adapted to, naturally oligotrophic areas^32^. In the Adriatic Sea, changes in nutrient budgets, increasing water temperatures and decreasing grazing pressure due to overfishing led to overgrowth of annual green algae (*Ulva* spp.)^33^, mostly at the expense of the endemic *Posidonia oceanica*^34^, whose meadows have been extensively reduced and which is virtually extinct in the Northern Adriatic^21,35^. The combination of enhanced temperatures and nutrient load also caused red tides and toxic algal blooms, followed by anoxia events that ultimately resulted in mass benthic mortalities in past decades (^21^ references therein). Therefore, these climate and human pressures may collectively pose a serious threat for the Adriatic Sea. Given the spatial heterogeneity in the distribution of these stressors and the multiple ways in which they can interact, their actual impacts on marine systems should be addressed on a case-by-case basis. Fortunately, our spatially-explicit assessments are on a fine enough scale to identify local areas of concern, even for small sub-regions within the Adriatic Sea. This is the case for the Northern Adriatic, where the highest nutrient concentrations and fishing pressure spatially co-occur (Fig. 4). Further studies on the interaction between these multiple stressors in these highly impacted areas are urgently required. They should be accompanied by effective management initiatives based on the redistribution of human impacts (e.g., fisheries) or site-specific management initiatives (e.g., wastewater treatment) so as to prevent collapses in the face of ongoing warming.

### Reductions in stressor levels are achievable

Reduction of human impacts, especially exploitation, habitat loss and pollution, has resulted in the slight recovery of ca. 10–50% of depleted marine populations and ecosystems worldwide^36^. Despite the magnitude of perturbations threatening the Mediterranean Sea and the lack of clear signs of successful recovery stories documented in the basin, our results leave room for optimism and suggest the SOS can provide a suitable framework for alleviating climate impacts on marine ecosystems through the management of interacting stressors operating at a more local scale. For instance, a partial decrease in nutrient concentrations in the Adriatic Sea over recent years^37,38^ (see also Fig. S2), likely due to changes in European environmental policies (see also^39^; and http://www.eea.europa.eu/themes/water/water-pollution/uwwtd/interactive-maps/urban-waste-water-treatment-maps-1 for wastewater treatment policies), illustrates how policies that aim primarily to clean up rivers (e.g. the EU Water Framework Directive and Urban Waste Water Treatment Directive) can provide major benefits for shallow seas. Although eutrophication levels in the Adriatic Sea are still relatively high (Figs 4 and S2) and marine ecosystems are not yet recovered^21,39^, these trends suggest that nutrient inputs can be effectively managed even in densely populated areas.

Management of human fisheries can also enable fish populations and marine communities to rebuild^40^. Although there is no evidence for fish stock recovery in the Mediterranean, fisheries and conservation management actions (including catch restrictions, gear modification, and management of marine areas for fisheries) in other marine ecosystems have resulted in the partial recovery of some previously collapsed fish stocks^41^. As foreseen by the SOS framework, management actions can therefore be implemented locally to effectively reduce human pressure and enhance ecosystem resilience to climate change, provided these management initiatives are based on adequate, spatially-explicit assessments on the distribution of climate and human impacts.

### Progress and the path ahead

Local or national agencies cannot single-handedly prevent climate change. However, they can enhance ecosystems’ resilience to climate impacts by managing stressors that operate at local or medium-scales^6,10^. The SOS framework aims to provide an “achievable” way for alleviating the consequences of climate change. However, effective solutions and suitable management measures depends on a better understanding of the spatial distribution of, and synergies between, climate and human impacts. We provide here one of the most spatially-explicit measurements available of the overlap between major stressors interacting synergistically in marine ecosystems. We have identified marine areas within the Mediterranean Sea where climate impacts are particularly severe and where local stressors should be reduced (e.g., through the spatial management of fisheries or reduction of nutrient loads) to enhance ecosystem resilience to climate change while the international community finds solutions for the global challenge posed by greenhouse gas emissions.

Management and conservation strategies may benefit from fine-scale, spatially-explicit assessments on the distribution of climate and human stressors such as those provided in this study. By definition, the SOS may incorporate as many dimensions as potential threats^6^ and provides also an adaptable framework that can be continuously updated by incorporating additional, spatially-explicit information on potential threats (e.g., invasive species, aquaculture or marine litter)^42–44^ or finer-scaled data on oceanographic features. However, there is still a considerable lack of knowledge regarding thresholds for stressors that cause ecosystem collapse when exceeded, and the manner in which these thresholds are lowered by additional stressors^45^. A thorough comprehension of the thresholds of interacting stressors and the patterns and mechanisms by which these stressors are impacting marine systems synergistically is still required for anticipating and preventing ecosystem collapses^6,45^.

## Methods

### Data sources for climate and human stressors

The NOAA (National Oceanic and Atmospheric Administration) high resolution (0.25° spatial resolution), optimum interpolation SST (V2; NOAA/OAR/ESRL PSD, Boulder, Colorado, USA; sourced online at http://www.esrl.noaa.gov/psd/, accessed in July 2017) was used to investigate trends and patterns in ocean warming in the Mediterranean Sea. This product provides daily information on SST for 35 complete years (1982–2016). It is derived from the Advanced Very High Resolution Radiometer (AVHRR) satellite data from the Pathfinder AVHRR SST dataset when available for September 1981 through December 2005, and from the operational Navy AVHRR Multi-Channel SST data for 2006 to 2016. The product also uses *in situ* data from ships and buoys, and includes a large-scale adjustment of satellite biases.

We also used a reanalysis of the Mediterranean Sea biogeochemistry (0.0625° spatial resolution) for 17 complete years (1999–2016). This is based on the OGSTM-BFM biogeochemical model and data assimilation of surface chlorophyll concentration. OGSTM-BFM was driven by physical forcing fields produced as output by the Med-Currents model. The ESA-CCI database of surface chlorophyll concentration estimated by satellite and delivered within CMEMS-OCTAC was used for data assimilation. This reanalysis provides monthly means of nutrient (phosphate and nitrate) concentrations, net primary production (NPP), dissolved oxygen concentrations (dO_2_), ocean pH and CO_2_ partial pressure (*p*CO_2_).

We used the fishing effort during the 2012–2017 period as a proxy of local impacts of human fisheries. Data on fishing effort were sourced online at the Global Fishing Watch website (http://globalfishingwatch.org/, accessed on August 2017). GFW estimates daily fishing effort by combining scores for all fishing vessels operating in the area. In turn, the fishing score model computes the probability that a vessel is fishing based on its AIS track data. Fishing is defined as the period that a vessel spends away from shore in which it is not transiting to and from the fishing grounds. This fishing effort data source combines information for the fishing activity of trawlers, longliners and purse seiners, and excludes the activity of artisanal fisheries (see^19^). Artisanal fisheries may contribute substantially to total fishing effort in the Mediterranean Sea, particularly in coastal areas^46^. Hence, our approach may not faithfully depict the fine-scale spatial distribution of fishing impacts. However, it is still useful for providing reliable information on the general patterns and distribution of fishing effort for relatively large regions such as the Mediterranean basin.

### Estimations of spatio-temporal patterns

Following^5^, pixel basis, least-square linear regressions for large (SST, pH and *p*CO_2_) and medium-scale stressors (phosphate and nitrate concentrations, NPP and dO_2_) were used for deriving the significance of temporal trends (α-value < 0.05) and their magnitudes (slopes; i.e. annual changes in target features) over recent decades. Temporal trends were calculated for the minimum, maximum and mean annual values of each variable. Although changes in environmental features may not follow linear trends, our simple, intuitive approach allowed us to map spatially-explicit information at the highest spatial resolution to date, on the significance of temporal trends and the magnitudes of observed changes over recent decades.

Climate (SST, pH and *p*CO_2_) and human stressors (phosphate and nitrate concentrations, NPP and dO_2_) were grouped and combined to obtain two cumulative, equally-weighted indexes. The slopes (a proxy to the magnitude of environmental changes) for those areas with non-significant trends (α-value > 0.05) were first set to 0. Absolute values for the slopes were then rescaled to the maximum value to make all variables comparable. These relative values (ranging from 0 – no change – to 1 – maximum recorded change –) were subsequently added on a pixel basis and rescaled again to the maximum value. In this way, we obtained two dimensionless indexes ranging from 0 (no change) to 1 (maximum change). Details on trends and slopes for single stressors are provided at Figs S1 and S2.

Spatially-explicit information on fishing effort was provided on a daily basis for 2012–2017. The overall assessment of the spatial distribution of fishing pressure was made by adding all the daily information, thus obtaining a single picture where marine areas particularly impacted by human fisheries over recent years were identified. This parameter was also rescaled to the maximum value to be combined with the two other cumulative indexes, thereby providing information about spatial heterogeneity in the magnitude of cumulative environmental changes, and areas where synergy between negative stressors can be expected.

## Electronic supplementary material

Supplementary Information

## Data Availability

Spatially explicit outputs produced in this work are hosted on the University of Barcelona digital repository (http://hdl.handle.net/2445/124833).
