# Supplementary material for: Spatial congruence between multiple stressors in the Mediterranean Sea may reduce its resilience to climate impacts
Source: Sci Rep. 2018 Oct 5;8:14871. doi: 10.1038/s41598-018-33237-w (PMC6173748; doi:10.1038/s41598-018-33237-w)

**Supplementary Information for:**

**Spatial congruence between multiple stressors in the Mediterranean Sea may reduce its resilience to climate impacts**

Francisco Ramírez, Marta Coll, Joan Navarro, Javier Bustamante, Andy J. Green

**Figure S1.** Significance and magnitude of observed long-term changes in the Mediterranean Sea’s global stressors: (a to c) sea surface temperature (SST; 1982-2016), (d to f) CO_2_ partial pressure (*p*CO_2_; 1999-2016) and (g to i) ocean pH (1999-2016). Colors represent the slopes of derived linear regressions for each single pixel; positive and negative trends are represented in red and blue scales, respectively. Gridded areas include those water masses with non-significant trends (α-value > 0.05).


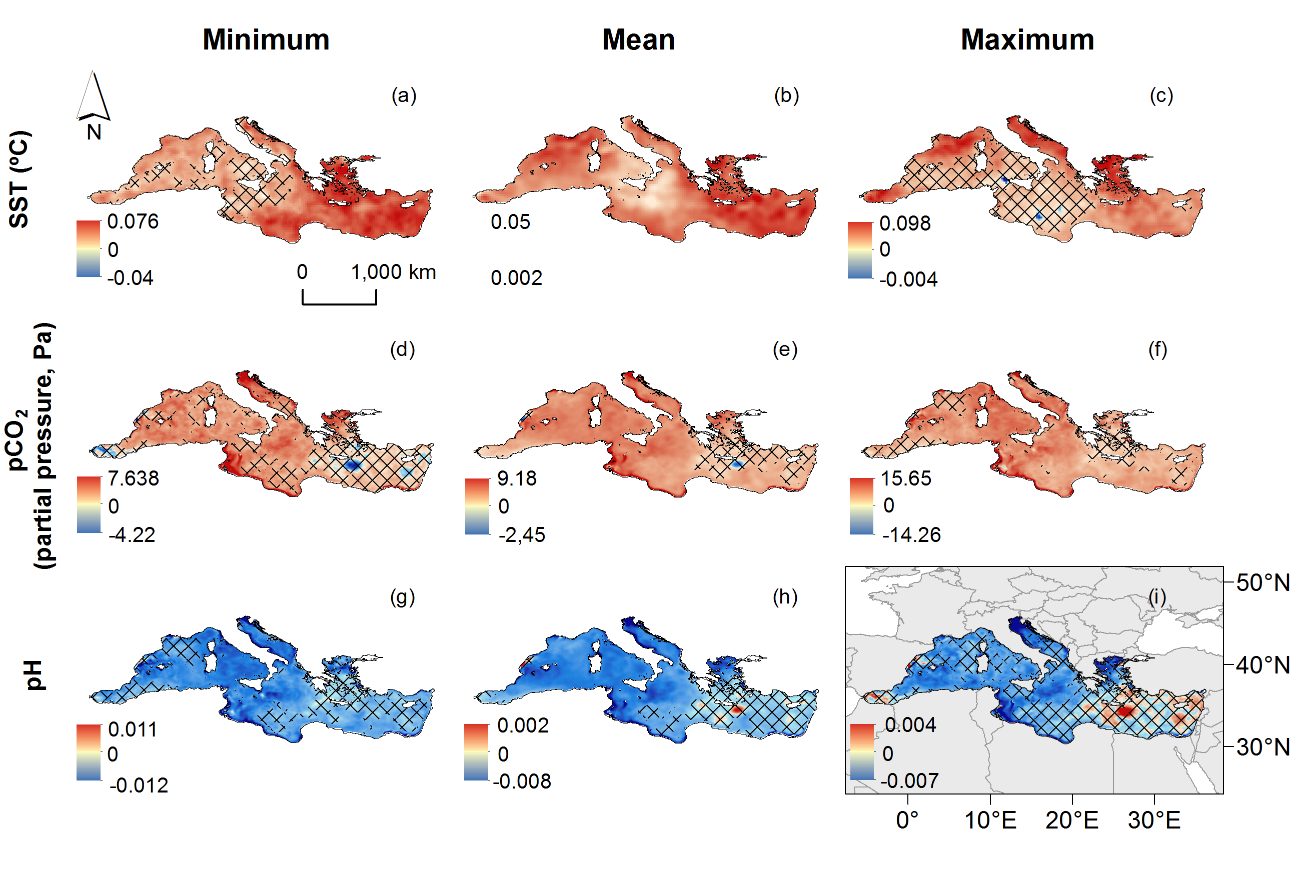


**Figure S2.** Significances and magnitude of observed long-term changes (1999-2016) in the Mediterranean Sea’s biochemistry: (a to c) phosphate concentration, (d to f) nitrate concentration, (g to i) Net Primary Production (NPP), (j to l) dissolved Oxygen (dO_2_). Colors represent the slopes of derived linear regressions for each single pixel; positive and negative trends are represented in red and blue scales, respectively. Gridded areas include those water masses with non-significant trends (α-value > 0.05).


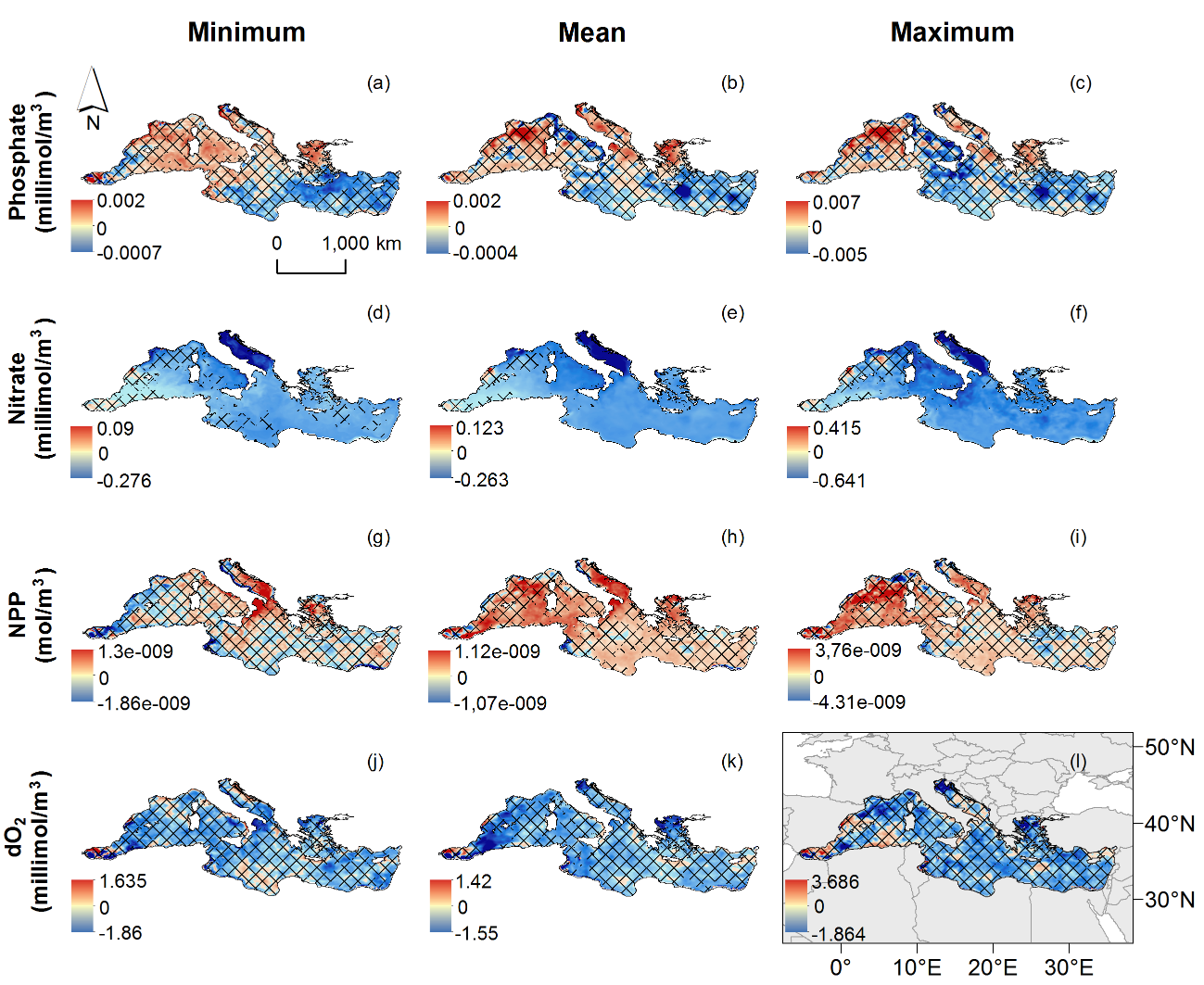

Supplement: Supplementary file 1 — Supplementary Information [file 41598_2018_33237_MOESM1_ESM.docx]
